# A comparison of laparoscopic procedures performed by novice medical students using 8K ultra-high-definition/two-dimensional and 2K high-definition/three-dimensional monitors

**DOI:** 10.1007/s00595-020-02215-z

**Published:** 2021-01-09

**Authors:** Tatsuya Shonaka, Chikayoshi Tani, Hiroyoshi Iwata, Masahide Otani, Kimiharu Hasegawa, Naoto Matsuno, Hiroyuki Furukawa, Akitoshi Yoshida, Yasuo Sumi

**Affiliations:** 1 Division of Gastrointestinal Surgery, Department of Surgery, Asahikawa Medical University, 2-1-1-1 Midorigaoka Higashi, Asahikawa, 078-8510 Japan; 2 Division of Hepato-Billiary-Pancreas Transplantation Surgery, Department of Surgery, Asahikawa Medical University, 2-1-1-1 Midorigaoka Higashi, Asahikawa, 078-8510 Japan; 3 Asahikawa Medical University, 2-1-1-1 Midorigaoka Higashi, Asahikawa, 078-8510 Japan

**Keywords:** 8K ultra-high-definition, Novice, 3D, Task performance

## Abstract

**Purpose:**

8K Ultra-high-definition (UHD) imaging has been developed in accordance with the progression of imaging technologies. We evaluated laparoscopic procedures performed by novice medical students using 2K/two-dimensional (2D), 2K/three-dimensional (3D) and 8K/2D monitors, with a particular focus on depth perception.

**Methods:**

Nine medical students were enrolled. They performed two tasks using 2K/2D, 2K/3D and 8K/2D monitors. In Task 1, they were asked to grasp three metal rods with forceps using each hand. In Task 2, they were asked to grasp a metal rod with forceps held in the right hand, pass the metal rod through a metal ring and transfer it to their left hand.

**Results:**

In Task 1, when performed with the dominant hand, the procedures performed using 2K/3D took a significantly shorter time than those performed using 8K/2D (*P* = 0.04). However, there was no significant difference among the three groups in the time required for procedures performed by the non-dominant hand. In Task 2, the procedure time with 2K/2D was significantly longer than that with 2K/3D or 8K/2D (*P* = 0.02).

**Conclusion:**

2K/3D showed superior utility to 8K/2D for performing forceps procedures using the dominant hand. However, when the movement of both hands was coordinated (“bi-hand coordination”), the laparoscopic procedures were performed almost as deftly with 8K/2D and 2K/3D.

## Introduction

The resolution of 8K ultra-high definition (UHD) imaging technology (7680 × 4320 pixels) is 16-fold higher than the current high-definition technology (HD; 1920 × 1080 pixels). 8K UHD has been developed in accordance with the advancement of new imaging technologies. Laparoscopic technology using 8K UHD/two-dimensional (2D) imaging has been available since 2000 [1]; however, the heavy weight and large size made it impossible to apply in the clinical setting. In 2014, the first laparoscopic cholecystectomy procedure using 8K UHD/2K laparoscopy was reported [2]. In the recent years, the application of 8K UHD laparoscopy has been extended to various operations, including the laparoscopic treatment of colorectal cancer [3].

Three-dimensional (3D) monitor technology has been developed for laparoscopy. 2K/3D imaging has been associated with a better laparoscopic surgical performance than a 2K/2D monitor [4, 5]. However, Yamada et al. [6] reported that while 2K/3D was useful for gastrointestinal surgeons, there was no marked difference in the performance between 2K/2D and 2K/3D for forceps procedures performed by pediatric surgeons. The advantages of 2K/3D and 4K/2D monitors in laparoscopic procedures performed by expert surgeons were evaluated [7], but whether or not 4K/2D imaging showed a better performance than 2K/3D imaging could not be demonstrated. Thus far, no reports have compared 8K/2D and 2K/3D laparoscopic procedures.

In the present study, we evaluated procedures performed by laparoscopic novice medical students using 2K/2D, 2K/3D and 8K/2D monitors, with a particular focus on depth perception.

## Methods

The study participants were medical students. We conducted a simple questionnaire survey that inquired about their laparoscopic procedure experience, age, dominant hand and other characteristics. Nomura et al. [8] reported trainees’ video game skill level or how highly they rated their laparoscopic skills. The subjects were randomly divided equally into three groups (2K/2D, 2K/3D and 8K/2D) and asked to perform two tasks. Enforcement was done in random order.

**Fig. 1:**
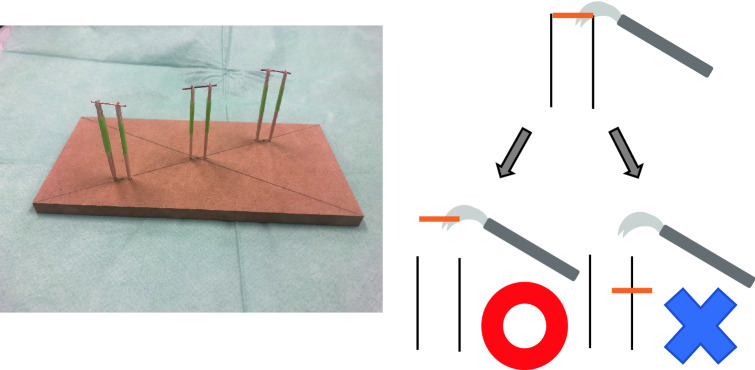
The apparatus of Task 1. We prepared metal rods that were placed on two rods at different distances (**a**). The metal rods were grasped with forceps using each hand (**b**). The act was successful when the participant grasped and lifted the metal rod. The students picked up metal rods in three different positions
- Task 2 (Fig. 2): A metal ring 1.5 cm in diameter was prepared. Touching a metal rod to the ring caused a light bulb to be illuminated. Subjects started by grasping a metal rod (3 cm in length) with forceps in their right hand. After slipping the metal rod through the metal ring, subjects then passed it to their left hand. We measured the time required to grab the metal rod with the left hand and release the forceps in the right hand. We evaluated the task time and number of times the bulb was illuminated. The purpose of Task 2 was to examine the coordinated movements of both hands (“bi-hand coordination”).

The 2K/2D and 2K/3D laparoscopes were from Olympus Co. (Tokyo, Japan), while the 8K/2D laparoscopes were from Kairos Co. (Tokyo, Japan). We used a training box to perform the tasks, which were originally created for this study. The size of the monitors was as follows: 2K/2D, 26 inches; 2K/3D, 31 inches; and 8K/2D, 72 inches. It is said that the distance at which the scanning line disappears is the most suitable viewing distance. A 2K HD monitor is best viewed at 3 times the vertical length of the monitor, and an 8K UHD monitor is best viewed at 0.75 times the vertical length of the monitor. These distances are the shortest critical distance for each screen at which the pixels constituting the screen cannot be identified; each of the three distances was recommended by the manufacturer. Thus, the distance between the participant and the monitor was fixed as follows: 2K/2D, 108 cm; 2K/3D, 150 cm; and 8K/2D, 75 cm.Task 1 (Fig. 1)

- Task 1 (Fig. 1): We prepared metal rods that were placed on two rods at different distances. The metal rods were grasped with forceps using one hand for each. The act was successful when the participant grasped and lifted the metal rod. The students picked up metal rods in three different positions. Each action constituted one set, and three sets were performed, with the average time evaluated. This procedure was performed with each hand. The time required to grip the rods and the number of successful grips were measured. The purpose of Task 1 was to study hand–eye coordination in simple one-hand procedures.

**Fig. 2:**
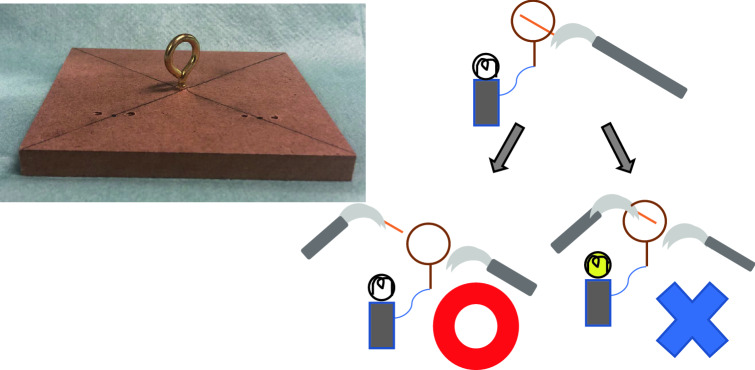
The apparatus of Task 2. We prepared a metal ring 1.5 cm in diameter (**a**). A metal rod was grasped with the right forceps. After slipping the metal rod through the metal ring, it was passed to the left hand (**b**). We measured the time taken to grasp the metal rod in the left hand and release the forceps in the right hand. If the forceps touched the metal ring during this act, the light bulb illuminated

Each participant performed each task three times, and the average times required for the performance of Tasks 1 and 2 were evaluated. Participants performed Tasks 1 and 2 using each monitor (2K/2D, 2K/3D and 8K/2D). After the task was finished, the students completed a five-part questionnaire survey that evaluated their impression of each monitor. This study was approved by the Asahikawa Medical University Research Ethics Committee (No. 19032). Written informed consent was obtained from the medical students.

The results were presented as the mean ± standard deviation within each group. All analyses were performed using the R software program (version 3.1.2) and the EZR software program [9]. The *χ*^2^ test or Fisher’s exact test was used for the analysis of categorical variables, and Student’s *t* test or the Mann–Whitney *U* test were used for the analysis of continuous variables. *P* values of < 0.05 were considered to indicate statistical significance. There were no reported conflicts of interest in this study.

## Results

Table 1 shows the background characteristics of the medical students. The median age was 24 years old. There were four males and five females. The dominant hand was the right hand in all cases. Approximately half of the medical students self-reported that they were good at playing the video games and driving, and were dexterous. Five of the medical students had experienced laparoscopic training, but each had experienced fewer than three training sessions.

**Table 1:** Backgrounds characteristics of the medical students

Variable		Value
Age (years)		24 (21–30)
Gender	Male Female	4 5
Interesting of laparoscopy	Yes No	6 3
Experience of laparoscopy	Yes No	5 4
Dominant hand	Right Left	9 0
Video game	Good Poor	5 4
Driving	Good Poor	3 6
Hand dexterity	Yes No	3 6

Unless otherwise mentioned, values are shown as the number of cases

**Fig. 3:**
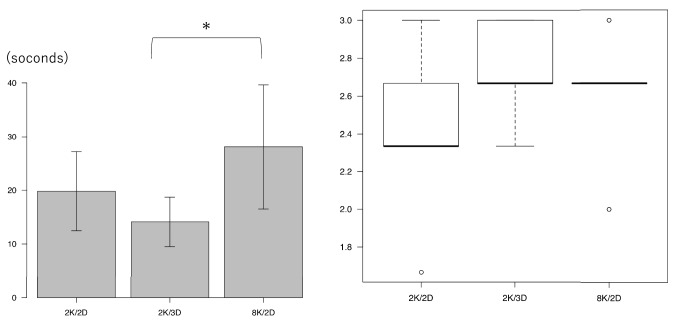
Task 1 performance using the right hand (dominant hand). **a** Task 1 performance time when using the right hand. *p < 0.05. **b** Number of successful grasps with the right hand

Figure 3 shows the students’ performance in Task 1 using their right hand (dominant hand). Figure 3a shows the time taken to perform Task 1 using the right hand: an average of 19.8 ± 7.4 s 14.1 ± 4.6 s and 28.1 ± 11.6 s when using the 2K/2D, 2K/3D and 8K/2D monitors, respectively. The time when using the 2K/3D monitor was significantly shorter than that when using the 8K/2D (*P* = 0.04). Figure 3b shows the number of successful grasps by the right hand in Task 1: an average of 2.4 ± 0.4, 2.7 ± 0.3 and 2.6 ± 0.4 when using the 2K/2D, 2K/3D and 8K/2D monitors, respectively. There was no significant difference among the three groups. The grasping of the metal rod with the forceps held in the dominant hand tended to be significantly easier to perform when using the 2K/3D monitor than when using the 8K/2D monitor.

**Fig. 4:**
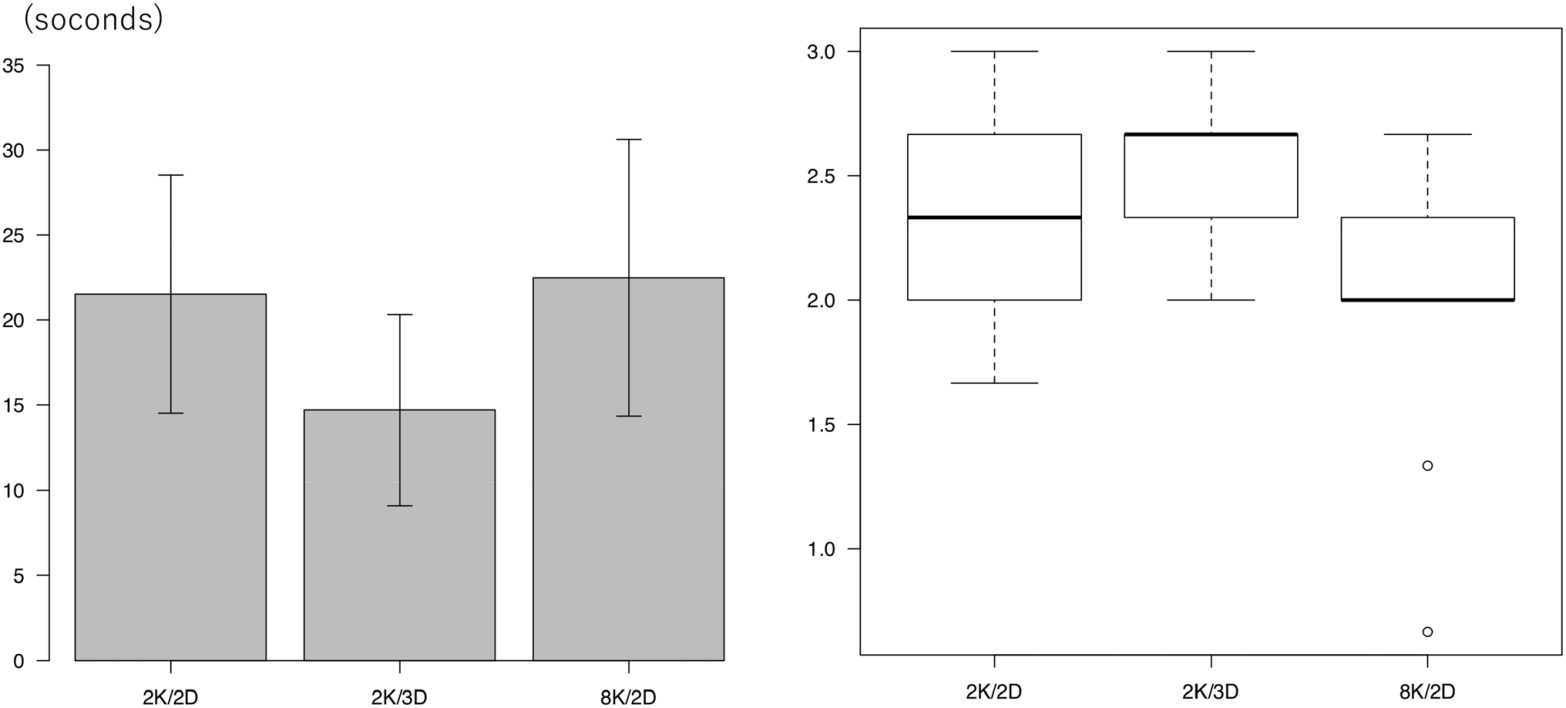
Task 1 performance using the left hand (non-dominant hand). **a** Task 1 performance time when using the left hand. **b** Number of successful grasps with the left hand

Figure 4 shows the students’ performance in Task 1 using their left hand (non-dominant hand). Figure 4a shows the time to taken perform Task 1 using the left hand: an average of 21.5 ± 7.0 s, 14.7 ± 5.6 s and 22.5 ± 8.1 s when using the 2K/2D, 2K/3D and 8K/2D monitors, respectively. Figure 4b shows the number of successful grasps by the left hand in Task 1: an average of 2.3 ± 0.5, 2.5 ± 0.4 and 1.9 ± 0.6 when using the 2K/2D, 2K/3D and 8K/2D monitors, respectively. There was no significant difference in the performance for grasping the metal rod with forceps in the non-dominant hand among the three groups.

**Fig. 5:**
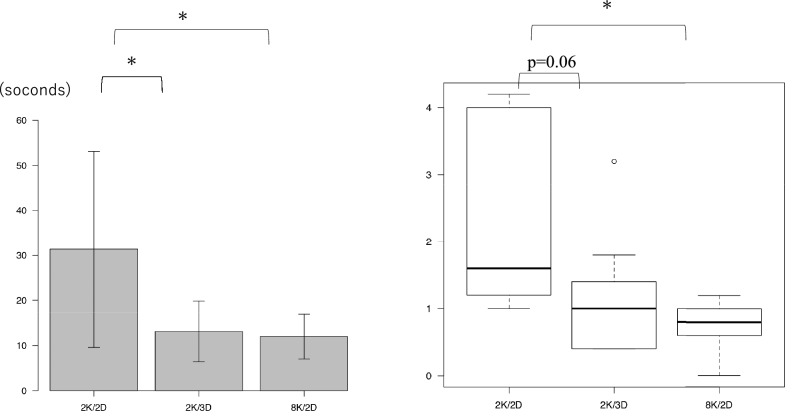
Task 2 results. **a** Time taken to perform Task 2. *p < 0.05. **b** Number of times the bulb was illuminated in Task 2. *p < 0.05

Figure 5 shows the results of Task 2. Figure 5a shows the enforcement time of Task 2: an average of 31.3 ± 21.7 s, 13.1 ± 6.7 s and 12.0 ± 4.9 s when using the 2K/2D, 2K/3D and 8K/2D monitors, respectively. The procedure time when using the 2K/2D monitor was significantly longer than that when using the 2K/3D and 8K/2D monitors (*P* = 0.02), while the procedure time when using the 2K/3D and 8K/2D monitors was almost the same. Figure 5b shows the number of times the light bulb was illuminated in Task 2: an average of 2.5 ± 1.4, 1.2 ± 0.9 and 0.8 ± 0.4 times when using the 2K/2D, 2K/3D and 8K/2D monitors, respectively. There was no significant difference when using the 2K/2D and 2K/3D monitors (*P* = 0.06). However, when using the 8K/2D monitor, the bulb was illuminated significantly fewer times than when using the 2K/2D monitor (*P* < 0.01). When the students performed the bi-hand coordination tasks using the 8K/2D monitor, the time taken to complete the task was shorter than when performing tasks with the 2K/2D monitor, and the number of procedural errors was lower.

Table 2 shows the results of the questionnaire survey after the tasks. The order of preference for the monitors (preferred to less preferred) was rated as follows: 2K/3D, 8K/2D and 2K/2D. The 2K/3D monitor was considered the best monitor for depth perception by the medical students.

**Table 2:** Results of the questionnaire survey after the tasks

	2K/2D	2K/3D	8K/2D
Total	2.9 ± 0.8	4.7 ± 0.5	3.9 ± 0.6
Depth perception	3.1 ± 1.4	4.7 ± 0.7	3.8 ± 0.7
Task 1 (right hand)	3.2 ± 1.0	4.7 ± 0.5	4.1 ± 0.3
Task 1 (left hand)	3.1 ± 1.2	4.8 ± 0.4	4.1 ± 0.6
Task 2	3 ± 1	4.8 ± 0.4	3.9 ± 0.8

## Discussion

The present study compared laparoscopic operations performed by novice medical students using 2K/2D, 2K/3D and 8K/2D monitors. There were two new findings in this study. First, we found that the 2K/3D monitor was superior to the 8K/2D monitor when performing forceps procedures with the dominant hand. However, there was no marked difference in the medical student performance with the 2K/3D and 8K/2D monitors when the forceps were held in the non-dominant hand. Second, when the medical students performed the coordinated movement of both hands (bi-hand coordination), the 2K/3D and 8K/2D monitors showed a similar performance and were both superior to the 2K/2D monitor.

Shiha et al. [10] reported that 3D monitors improved hand–eye coordination in comparison to 2D monitors. The report also showed that 3D monitors improved the depth perception in comparison to 2D monitors. A 3D monitor allows for the perception of depth, so hand–eye coordination can easily be achieved with the dominant hand. Our questionnaire results showed that the medical students evaluated the 3D monitor highly with regard to depth perception. In our study, the performance in procedures in which the forceps were held in the non-dominant hand was not very good. These novice medical students were not considered practiced in performing forceps procedures with their non-dominant hand. Although some reports have described the effectiveness of 3D monitors when used by novices [11–13], few have compared the performance of the dominant and non-dominant hands. This result proves that 3D monitors were effective for novice medical students specifically because they were able to achieve hand–eye coordination of the forceps in the dominant hand. Our finding that 8K/2D was inferior to 2K/3D is interesting. The medical students had difficulty adjusting the forceps near the target. It seems that an excessive magnifying effect was obtained when performing these actions using a large 8K/2D monitor. The manipulation velocity of the forceps on the large 8K/2D monitor may be misrepresented compared with that seen on 2K/2D and 2K/3D monitors. Therefore, for these novice medical students, the difference in speed on the monitor may have led to operational difficulties. Further studies are warranted to examine the relationship between the monitor size and manipulation velocity on different screen sizes for an objective evaluation.

When the medical students performed the coordinated movement of both hands (bi-hand coordination), the 2K/3D and 8K/2D monitors showed superior performance to the 2K/2D monitor. In the 2K/3D and 8K/2D monitors, the task performance of bi-hand coordination were almost the same. Of note, the 2K/3D task performance were more efficiently with one hand, regardless of whether it was the dominant of non-dominant hand. When the students performed tasks with both hands, any advantage was lost, and the performance was similar when using the 2K/3D and 8K/2D monitors.

Carlos et al. [14] showed that pre-training in hand–eye coordination with both the dominant and non-dominant hands shortens the learning curve. When the 8K UHD image quality was utilized with both hands, decent hand–eye coordination was achieved, and “pseudo 3D imaging” was obtained. However, according to the Task 1 questionnaires, 8K/2D was generally considered inferior to 2K/3D. Saseem et al. [15] reported that novices trained in a 3D environment failed to transfer their skills to a 2D environment. When participants become used to a 3D environment, novices might find it difficult to perform tasks using 2D monitors. However, our results suggest that when surgical operations using both hands are performed, a similar degree of performance might be obtained using 8K/2D and 2K/3D monitors.

Several limitations associated with the present study warrant mention. This study was a comparative study among 2K/2D, 2K/3D and 8K/2D monitors, and evaluations were made based on simple procedures. Furthermore, in this study, the 8K monitor showed much more visual information than the 2K monitor; however, we did not evaluate the influence of the visual information.

In conclusion, we examined laparoscopic procedures performed by novice medical students using 2K/2D, 2K/3D and 8K/2D monitors. 2K/3D was superior to 8K/2D in forceps procedures performed with the dominant hand. However, when the coordinated movement of both hands (“bi-hand coordination”) was required, the performance using the 8K/2D monitor was almost the same as that using the 2K/3D monitor.

## References

[1] Sugawara M, Kanazawa M, Mitani K (2003). Ultrahighdefinition video system with 4000 scanning lines. SMPTE Motion Imaging J.

[2] Yamashita H, Aoki H, Tanioka H, Mori T, Chiba T (2016). Ultra-high definition (8K UHD) endoscope: our first clinical success. SpringerPlus.

[3] Ohigashi S, Takeda T, Shimada G, Kubota K, Sunagawa H, Kishida K (2019). Fruitful first experience with an 8K ultra-high-definition endoscope for laparoscopic colorectal surgery. Asian J Endosc Surg.

[4] Mashiach R, Mezhybovsky V, Nevler A, Gutman M, Ziv A, Khaikin M (2014). Three-dimensional imaging improves surgical skill performance in a laparoscopic test model for both experienced and novice laparoscopic surgeons. Surg Endosc.

[5] Ashraf A, Collins D, Whelan M, O’Sullivan R (2015). Balfe P Three-dimensional (3D) simulation versus two dimensional (2D) enhances surgical skills acquisition in standardised laparoscopic tasks: a before and after study. Int J Surg.

[6] Yamada K, Murakami M, Yano K, Baba T, Harumatsu T, Onishi S (2019). Impact and characteristics of forceps manipulation of 3D in laparoscopic hepaticojejunostomy mimicking disease specific simulator Comparison with expert to trainee. J Laparoendosc Adv Surg Tech.

[7] Harada H, Kanaji S, Hasegawa H, Yamamoto M, Matsuda Y, Yamashita K (2018). The effect on surgical skills of expert surgeons using 3D/HD and 2D/4K resolution monitors in laparoscopic phantom tasks. Surg Endosc.

[8] Nomura T, Miyashita M, Shrestha S, Makino H, Nakamura Y, Aso R (2018). Can interview prior to laparoscopic simulator training predict a trainee's skills?. J Surg Educ.

[9] Kanda Y (2013). Investigation of the freely available easy-to-use software 'EZR' for medical statistics. Bone Marrow Transplant.

[10] Sinha RY, Raje SR, Rao GA (2017). Three-dimensional laparoscopy: principles and practice. J Minim Access Surg.

[11] Chiu CJ, Lobo Prabhu K, Tan-Tam CC, Panton ON (2015). Meneghetti A Using three-dimensional laparoscopy as a novel training tool for novice trainees compared with two-dimensional laparoscopy. Am J Surg.

[12] Smith R, Day A, Rockall T, Ballard K, Bailey M, Jourdan I (2012). Advanced stereoscopic projection technology significantly improves novice performance of minimally invasive surgical skills. Surg Endosc.

[13] Alaraimi B, El Bakbak W, Sarker S, Makkiyah S, Al-Marzouq A, Goriparthi R (2014). A randomized prospective study comparing acquisition of laparoscopic skills in three-dimensional (3D) versus two-dimensional (2D) laparoscopy. World J Surg.

[14] Molinas CR, Binda MM, Campo R (2017). Dominant hand, non-dominant hand or both? The effect of pre-training in handeye coordination upon the learning curve of laparoscopic intra-corporeal knot tying. Gynecol Surg.

[15] Poudel S, Kurashima Y, Watanabe Y, Ebihara Y, Tamoto E, Murakami S (2017). Impact of 3D in the training of basic laparoscopic skills and its transferability to 2D environment: a prospective randomized controlled trial. Surg Endosc.

